# Material fictions: Comparing physically based renderings and generative AI images through material perception

**DOI:** 10.1167/jov.26.3.7

**Published:** 2026-03-16

**Authors:** Yuguang Zhao, Jeroen Stumpel, Huib de Ridder, Jan Jaap R. van Assen, Maarten W. A. Wijntjes

**Affiliations:** 1 Perceptual Intelligence Lab, Faculty of Industrial Design Engineering, Delft University of Technology, Delft, the Netherlands; 2 Department of History and Art History, Utrecht University, Utrecth, the Netherlands

**Keywords:** material perception, generative AI, ordinal embedding, BRDF rendering, ControlNet

## Abstract

Generative artificial intelligence (AI) models unlock new ways to create images, emerging as a new medium alongside paintings, photographs, physically based renderings (PBR), etc. Generative AI images can be perceptually convincing without being physically plausible, allowing to investigate the boundaries of visual perception. This study examines whether generative AI images adhere to a medium-independent perceptual space converged from previous studies. We compared the perceptual similarity of images from three generative AI models against a bidirectional reflectance distribution functions (BRDFs) PBR image dataset, using human similarity judgments. In experiment 1, we used the text descriptions of 32 materials (e.g., blue acrylic) from the Mitsubishi Electric Research Laboratories (MERL) BRDF dataset, prompting two text-to-image models, DALL-E 2 and Midjourney v2, to generate 32 sphere-shaped stimuli per model. Perceptual spaces derived from similarity judgments revealed that both AI models resulted in two-dimensional spaces whereas the MERL space was confined to one dimension, probably owing to a lack of surface texture. These unrelated perceptual spaces suggest the AI models generated unique and different images from identical text prompts. In experiment 2 we used the text-to-image model Stable Diffusion v1.5 with ControlNet for additional depth-map constraints. Using the same 32 descriptions, we generated 3 sets using 3 different depth maps. The three resulting perceptual spaces are all two-dimensional, exhibiting high similarity, indicating a robust and non-random structure. They also show a similar structure to the MERL space and perceptual spaces from other material studies using photographs, PBR, and depictions, suggesting AI-generated imagery may indeed be used as a new medium to explore material perception.

## Introduction

We are surrounded by a large variety of materials that visually display various properties, for example, physical properties such as hardness, roughness, or viscosity (Fleming, 2017). Although the natural environment already contains a large variety of materials, the contemporary (built) environment also includes an increasing number of manufactured materials. This apparent material complexity is an interesting topic for the study of visual perception, because humans likely reduce this complexity by grouping materials into categories, enabling them to estimate properties such as those mentioned over a large variety of materials (Fleming, 2017; Schmidt, 2019). Moreover, we encounter an ever-increasing number of *images* of materials, such as a photo of a glass building facade on a phone screen, a computer-rendered rock in a game on a laptop, or a painting depicting ocean water.

There are various approaches to understanding human material perception. Most studies use images of materials instead of the actual physical objects. Having control over physical characterizations of materials has been the norm in material perception studies over the past decades, either using photos in combination with physical measurements or using physics-informed computer renderings.

Images may have different properties, characters or styles, both among themselves, and when compared with material perception in actual environments. Therefore, it may be important to probe possible differences between different varieties of generated images of materials. Over the past decades, computer-generated imagery (CGI) has become a dominant technique in the movie and gaming industries, and rendering innovations have been developed in tandem with insights from perception research (Khan, Reinhard, Fleming, & Bülthoff, 2006; Thompson, Fleming, Creem-Regehr, & Stefanucci, 2011; Vangorp, 2009). When studying specific material properties, the complex relationship between visual cues, and material perception often requires restricting the study to a single material category, or sometimes even maintaining a constant object shape. For example, to understand gloss, Wills, Agarwal, Kriegman, and Belongie (2009) used computer-rendered bunnies with different bidirectional reflectance distribution functions (BRDFs), and Ferwerda, Pellacini, and Greenberg (2001) used rendered spheres as stimuli. These studies used computer renderings because they afford precise control over the various distal (or world) parameters that define materials, such as reflectance characteristics.

At the same time, it appears possible to investigate cue–perception relations in uncontrolled stimuli as shown in the glossiness study by Di Cicco, Wijntjes, and Pont (2019). They investigated painterly practice and defined cue intensities by measuring various image properties such as contrast and blur. Using art images instead of renderings has the added value that a certain pictorial approach of the maker is automatically incorporated, which may reveal additional insights into mechanisms of material perception. Furthermore, paintings are made on surfaces such as canvas, not in a physics rendering engine. As a result, painters are not limited by the rigidity of physically based rendering algorithms (Cavanagh, 2005).

As anyone living at the time of our study must have noticed, a new medium has become available: generative artificial intelligence (AI). Although synthetic textures have already existed for several decades (Efros & Leung, 1999; Heeger & Bergen, 1995; Portilla & Simoncelli, 2000), deep neural networks revolutionized the production of images with the invention of generative adversarial networks (GANs) (Goodfellow et al., 2014). Instead of being trained to only recognize and classify depicted objects such as in AlexNet (Krizhevsky, Sutskever, & Hinton, 2012), this new type of network made it possible to expand the kinds of classifications, for example, classifying whether an image is a photo or not. This architecture resulted in rather photorealistic images albeit with a certain uncanniness. Interestingly, this type of bug is often regarded by artists such as Mario Klingeman and Helene Sarin as a positive feature to help creating aesthetically pleasing images (Hertzmann, 2020; Wang, Bylinskii, Hertzmann, & Pepperell, 2020). Combining text and images became the next significant innovation, for example by using the CLIP model (Radford et al., 2021). This resulted in various generative image synthesizers based on so-called prompts, text describing what (and how) the image should depict. Around 2022, various platforms started their online services of text-based image generation (e.g., DALL-E and Midjourney) or released their model (Stable Diffusion).

Generative AI pictures conceptually resemble paintings as the generation takes place in the picture plane, that is, the RGB matrix and canvas, respectively. In contrast, for computer rendering and photography there is always a three-dimensional (3D) source of which the image is the projection. Generative AI depictions do not originate from distal scene properties and, consequently, cannot be directly linked to physical parameters. However, it is likely that there are latent space correlates for various visual phenomena (Goetschalckx, Andonian, Oliva, & Isola, 2019; Liao, Sawayama, & Xiao, 2023) comparable to those found in face editing (Shen, Gu, Tang, & Zhou, 2020). Unlike rendering, there is no direct link to physical parameters, which means that generative AI depictions are not limited by the laws of physics, just like paintings, drawings, and so on. This is important, because the human visual system is also not bound to the laws of physics but rather may use its own intuitive physics (Cavanagh, 2005; McCloskey, 1983; Piloto, Weinstein, Battaglia, & Botvinick, 2022; Garrido et at., 2025; Battaglia, Hamrick, & Tenenbaum, 2013) to model the outside world.

For vision scientists, generative AI imagery offers interesting opportunities. Without artistic training, it is possible to create stimuli that are unreal but still perceptually convincing. This allows researchers to probe how the visual system generalizes from all things and stuff previously seen toward unseen, novel stimuli. A central property of the visual system is to make sense of previously unseen events, objects, and environments. Generative AI can create such new stimuli and thereby investigate the boundaries of what is perceptually interpretable. For material perception, this approach opens up possibilities to create physically implausible stimuli with which we can probe the sensitivity of the visual system to violations of physical inconsistencies. Before entering this largely unexplored territory, we decided to first perform a more traditional material perception experiment where we could compare the generative AI stimuli with a certain standard, in our case a BRDF dataset. A scientifically relevant and established context for this comparison is that of perceptual material spaces, as we will outline in the next paragraph.

The ability of generative AI to create photorealistic images raises fundamental questions about how these models represent material properties and how those representations align with human perception and underlying processing structures. Before the rise of generative AI images, material perception studies seem to lead to a medium-independent perceptual space (Zhang, de Ridder, Barla, & Pont, 2019), no matter whether the stimuli being used are real-world photos (Fleming, Wiebel, & Gegenfurtner, 2013), CGI renderings (Zhang et al., 2019), paintings (van Zuijlen, Pont, & Wijntjes, 2020) or even verbal descriptions (Fleming et al., 2013). In this study, we investigated how this medium-independent material perception space relates to this new medium, generative AI. Quantifying perceptual spaces can be used to explore core dimensions in material perception (Schmidt et al., 2022), but also in style perception (Zhao, Stumpel, de Ridder, & Wijntjes, 2023), and in many other fields where an a priori structure is lacking. The traditional method to create a perceptual space is multidimensional scaling (MDS) (Mead, 1992), where observers are asked to rate the difference between each pair of stimuli and the resulting scores are transformed into distances in a multidimensional space. To avoid individual scaling differences, various other methods have been developed, for example, the ones that make use of triplets where the observer is asked to select the two most similar stimuli per trial. This ordinal information can then be processed with, for example, (landmark) MDS (De Silva & Tenenbaum, 2004; Zhao et al., 2023) or specific neural networks (Hebart, Zheng, Pereira, & Baker, 2020). These two methods address the challenge of the quickly increasing number of possible triplets—scaling cubically with the number of stimuli—by applying various techniques to reduce the required minimum number of triplets. A relatively new and promising method that seems to require the least data for generating robust perceptual space reconstructions is soft ordinal embedding (SOE). Originated from machine learning, the goal of SOE is to find an embedding (perceptual space) that maximizes the number of consistent triplets (Haghiri, Wichmann, & von Luxburg, 2020; Künstle, von Luxburg, & Wichmann, 2022; Künstle & von Luxburg, 2024; Terada & Luxburg, 2014).

In this study, we chose an established BRDF material dataset, Mitsubishi Electric Research Laboratories (MERL) dataset (Matusik, 2003) as our starting point. Each material from MERL comes from real-world measurements of physical materials and contains text description of the material (e.g., blue acrylic). The text descriptions are used as prompts for AI image generation. We acknowledge that the BRDF labels were not intended to be used as prompts. However, they were the verbal descriptions MERL dataset authors used to label those material images. We used the exact labels as prompts for the AI models for two following reasons: 1) we were interested what images the AI models would create from those material labels, and 2) because we believe that studying the exact effect of various prompt engineering variables is beyond the scope of the current study, we choose to stick to the literal labels. As the reader will notice, this sometimes led to unexpected results, which we consider acceptable given our decision to limit prompt optimization to keep the focus of the study on material perception. As for the shape, more specifically, we choose the stimuli by Lagunas et al. (2019) who used the BRDFs of MERL to generate a large dataset. Lagunas et al. (2019) first collected human similarity judgments for a subset of the stimuli and then used these data to train a deep learning model that can measure (predict) the appearance similarity between different materials. One of the shapes they used was a sphere, being one of the very few geometric shapes that is unambiguously captured by text, which is the input for our stimulus generation. A cube would also be possible, but objects with a tessellated structure can fail to evoke correct reflectance properties (Vangorp, Laurijssen, & Dutré, 2007). Moreover, although a cube seems to be a very simple shape to describe with text, it can produce different resulting images depending on its orientation and viewing distance. In our second experiment, however, we explored an alternative technique for generating more complex shapes using ControlNet (Zhang, Rao, & Agrawala, 2023). The comparison between generative AI imagery and the established material frameworks will expand our understanding of how AI models generate materials and their potential as a tool to expand our knowledge of visual perception.

## Experiment 1: Influence of generative AI model

Experiment 1, conducted in 2022, investigated two popular text-to-image generative AI models, DALL-E 2 (Ramesh, Dhariwal, Nichol, Chu, & Chen, 2022) and Midjourney v2 (https://www.midjourney.com/). We compared images generated from these two models with a computer graphics rendering dataset by Lagunas et al. (2019), who rendered spheres with various BRDFs from the MERL dataset (Matusik, 2003) under various light probes (Debevec, 2008). For the generative AI models, the only constraint of the output images is the text description (in contrast with the image constraints we used in Experiment 2).

### Methods

#### Stimuli

**Figure 1.**
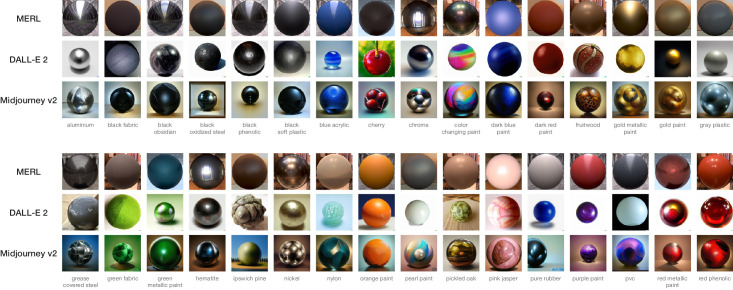
An overview of all stimuli used in Experiment 1. The word(s) below each group of images are the text descriptions from the MERL dataset. We used the same descriptions as prompts for the AI models but added the word ‘sphere.’ (i.e., ‘aluminium’ becomes ‘an aluminium sphere’).

We used three sets of images, each containing 32 comparable materials. An overview is shown in Figure 1. The first set, MERL, is a BRDF dataset based on real-world measurements. We chose to include images with different environment maps to ensure diversity and anticipate on a variety of ‘lighting’ settings in the generative AI stimuli. Six environment maps were used, Uffizi, Grace, Pisa, Ennis, Glacier and Doge (Debevec, 2008), for 10 images, 7 images, 7 images, 4 images, 3 images, and a single image, respectively. Each material comes with a text description (e.g., ‘blue acrylic’) as specified by the BRDF name in the original MERL dataset. The other two sets were generated with generative AI models, DALL-E 2 and Midjourney v2. Each set contains 32 comparable materials; we used the BRDF names as prompts to generate the images. To control the shape, we added the word ‘sphere’ in the text prompt. Examples of prompts are ‘a blue acrylic sphere’ and ’a chrome sphere.’ Note that participants only saw the images of materials, not the text description. Moreover, it is important to note that the BRDF label name, together with a constraint on object shape ‘sphere,’ was the only information used to generate the AI stimuli. Unlike the MERL stimuli, the generative AI stimuli were not associated with an explicit environment map, because they were generated directly from learned image statistics rather than rendered using a physical illumination model.

#### Procedure

**Figure 2.**
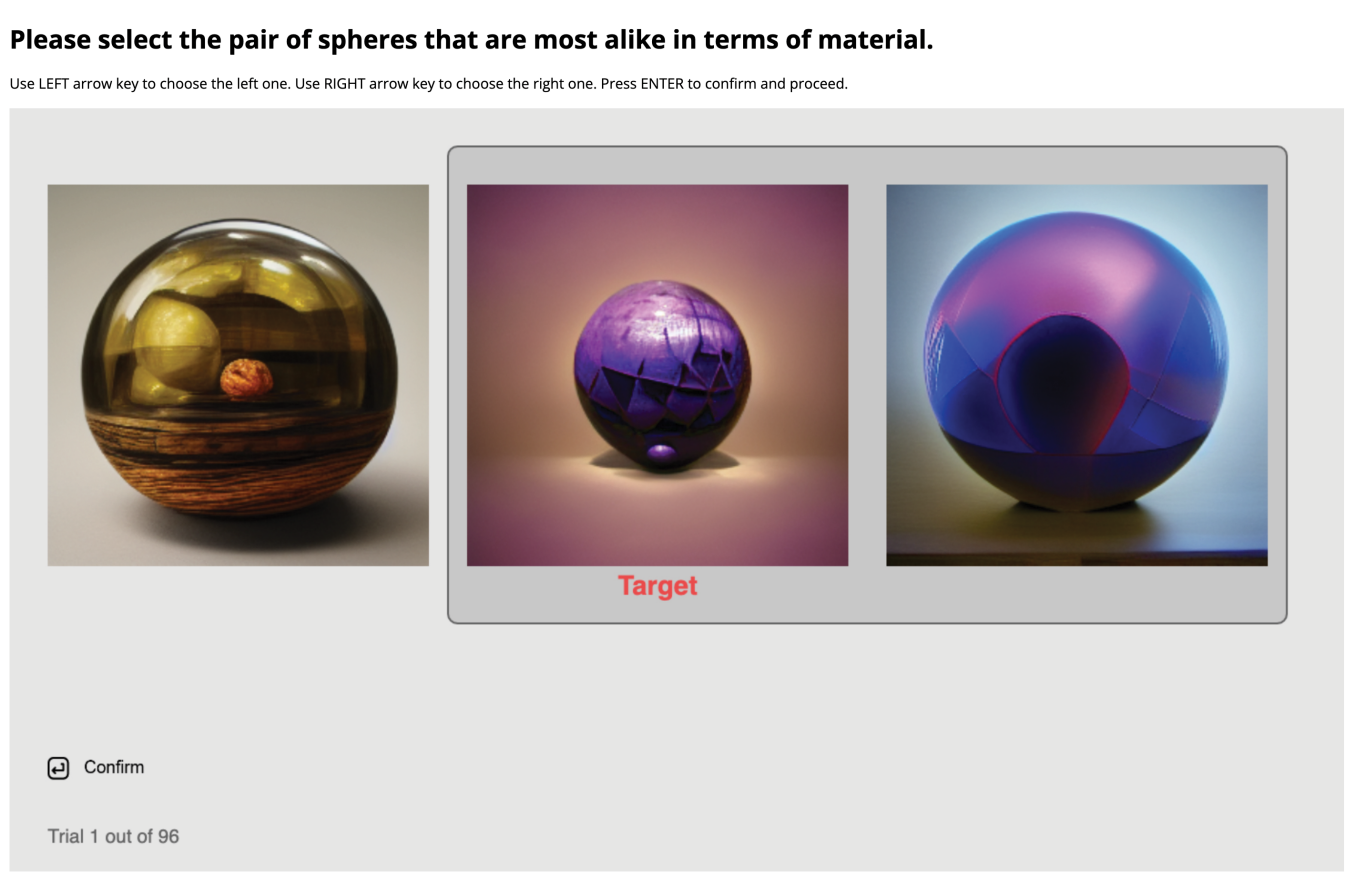
Experiment interface of Experiment 1, showing images from Midjourney v2. Participants were shown three images of different materials, where both the selection and order were randomized. Their task was to select either the left image or the right image that is most alike the center one (target) in terms of material. Participants could use the LEFT and RIGHT arrow keys to select their choice by sliding the window, then press RETURN to confirm and proceed to the next trial.

Because we are interested in the perceptual spaces from these three image sets, we chose a similarity judgment task. We conducted three online experimental sessions, one for the MERL, one for the DALL-E 2, and one for the Midjourney v2 image sets. Each session contained 96 trials after 15 practice trials for participants to become familiar with the concept and operation. In each trial, participants were presented with a triplet of images; both the selection and order were randomized. The center stimulus was set as the target, the task was to select either the left or the right one as the one most similar to the center target^1^ in terms of material. Figure 2 shows the experiment interface. Participants could use the left and right arrow keys to indicate their choice, then use the ‘return’ key to both confirm and proceed to the next trial. One benefit of the triplet judgment task (over similarity rating) is the ability to scale up the experiment by combining data across multiple participants, without the issue of different internal scaling (O’Hare, 1976; Linde, 1975). This advantage makes triplet method better suited for crowd-sourcing studies (Heikinheimo & Ukkonen, 2013; Li, Endo, & Kashima, 2021; Tamuz, Liu, Belongie, Shamir, & Kalai, 2011).

#### Participants

We recruited 150 unique participants for Experiment 1, 50 participants for each session. A server issue caused some data loss. Eventually, we recorded 45 (for MERL), 35 (for DALL-E 2), and 40 participants (for Midjourney v2) for 3 sessions. All participants received compensation regardless of their data being recorded. All participants were recruited from Prolific (www.prolific.com). The following prescreen criteria were used: 1) approval rate 95% to 100%, 2) number of previous submissions 100 to 1,000, 3) highest education level completed higher than high school, 4) fluent in English, 5) from the United States or the UK, and 6) exclude participants from our previous studies. Note that criteria 3 through 5 were used to make sure participants could understand the instructions properly without language barrier. The experiment was conducted in agreement with the Declaration of Helsinki and approved by the Human Research Ethics Committee of the Delft University of Technology. All data were collected anonymously.

#### Data analysis

We used SOE (Haghiri et al., 2020; Künstle et al., 2022) to convert the triplet data into perceptual embeddings. Compared with other similar scaling methods, this method does not necessitate the data of all possible triplets, but rather requires only 2*dn*log_2_*n*, where *d* is the estimated number of dimension(s) and *n* is the number of stimuli, without losing accuracy. Based on previous research, we expected at least a two-dimensional (2D) solution. To anticipate a possible five-dimensional or six-dimensional solution (1,920 triplets), we aimed for 50 participants (50 × 96 trials = 4,800 triplets). Having more triplets also improves the accuracy of the results.

After getting the embeddings from the SOE algorithm (Künstle & von Luxburg, 2024), we first conducted Procrustes analysis for embeddings with the same dimensionality, where scaling, rotation, translation, and reflection were applied separately to the DALL-E 2 and Midjourney v2 embeddings so that both embeddings were optimally aligned with the MERL embedding. After alignment with Procrustes analysis, the embeddings can be compared more directly and more easily. Then we calculated the canonical correlations between the three embeddings, quantifying the similarities between them. Besides the overall correlation coefficient and its significance, the calculation also yields weights on the dimensions, indicating the importance of each dimension, thus helping us to interpret the canonical correlations.

### Results

**Figure 3.**
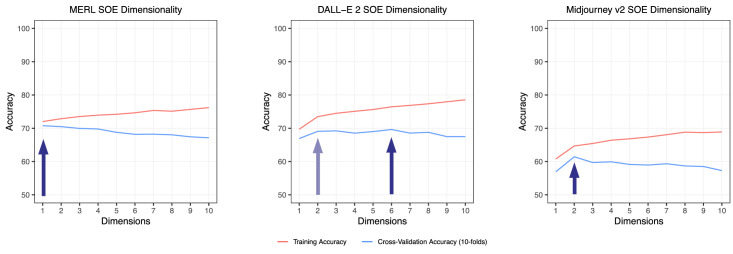
The dimensionality of MERL, DALL-E 2, and Midjourney v2 embeddings. The blue cross-validation curves show the overall performance of the fit. The peak of the cross-validation accuracy curve, indicated by the blue arrows, stands for its optimal dimensionality. Error bars are not included because the variance between folds is very small.

**Figure 4.**
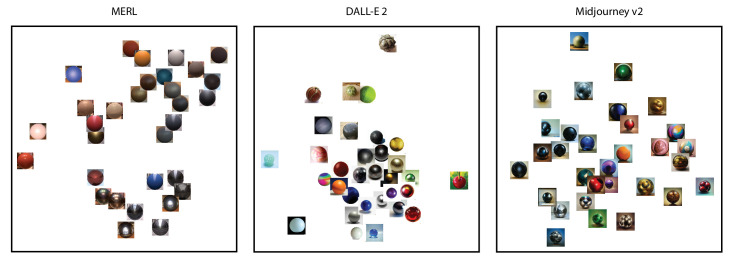
The 2D embeddings of MERL, DALL-E 2, and Midjourney v2. Both DALL-E 2 and Midjourney v2 embeddings are aligned with MERL after Procrustes analysis. The *y*-axis of the MERL embedding is positively associated with the 1D solution for MERL.

First, we determined the dimensionality by looking at the cross-validation accuracies, as shown in Figure 3. The blue curves denoting the 10-fold cross-validation accuracy measured the percentage of triplet data that can be correctly predicted by the embedding. To this end, the data were split multiple times into training and validation data to exhaustively use the entire dataset for both training and validation in a systematic manner. A higher value indicated a better fit. The peak of a cross-validation curve indicated its optimal dimensionality. In theory, this method would prevent both underfitting and overfitting, because decreasing or increasing the number of dimensions will not provide any accuracy gain. Our data suggested a 1D solution for MERL materials, a 2D or six-dimensional solution for DALL-E 2 materials, and a 2D solution for Midjourney v2 materials. For direct comparison, we plotted the 2D embeddings for all three spaces. Figure 4 shows the three 2D embeddings after Procrustes analysis with the DALL-E 2 and Midjourney v2 embeddings aligned with MERL embedding. From observation, they show low similarity even after the Procrustes alignment.

Second, we applied canonical correlation analysis to quantify possible similarities between these embeddings. Table 1 shows the results of this analysis. The canonical weights indicated how much each dimension contributes to the overall correlation. The three correlation coefficients appeared to be relatively low, suggesting hardly any relation between these three embeddings. This underscores the visual inspection of Figure 4. The only case that yielded a significant correlation was between the MERL and Midjourney v2 embeddings, with a relatively low correlation coefficient (*r* = 0.550, *p* < 0.05). As for the weights, the *y* axis (0.691) from the MERL embedding contributed slightly more than the *x* axis (−0.584), where the *x* axis (0.907) from the Midjourney v2 embedding contributed much more than the *y* axis (0.437) to the overall correlation. This may be attributed to the corresponding presence of metallic, glossy stimuli in the lower half of the MERL embedding and the lower left quarter of the Midjourney v2 embedding.

**Table 1.** Canonical correlation results for Experiment 1.

			Canonical weights			
Space1-space2	Correlation coefficient	*p* value	Space1-X	Space1-Y	Space2-X	Space2-Y
MERL-Dalle	0.283	0.616	−0.071	0.968	−0.513	0.898
MERL-Midj	0.550	0.017	−0.584	0.691	0.907	0.437
Dalle-Midj	0.073	0.997	−0.996	−0.005	−0.705	−0.722

The canonical weights indicate how much each dimension contribute to the overall correlation.

### Discussion

Looking at the dimensionality plot from Figure 3, MERL and the two AI models yielded different dimensionalities. Both DALL-E 2 and Midjourney v2 have higher dimensions than MERL; that is, they need more dimensions to explain the perceptual differences between the stimuli. A possible explanation is the difference in surface texture: being a BRDF material dataset, MERL has no surface texture, where both DALL-E 2 and Midjourney v2 have texture on the surface. The additional information of surface texture increases stimulus complexity which may cause an increase in dimensionality.

Both the different number of dimensions and low correlations as shown in Table 1 suggest low similarity among these perceptual spaces. Beside the difference in surface texture, another explanation for this low similarity might be related to semantics. The MERL dataset comes from measurements of real-world materials, with text descriptions. For AI-generated images, however, the text prompts are the starting point. The output images are the interpretation of the described material by the AI models. In some cases, the interpretation from the AI models is rather literal. For example, the three datasets have very different appearances of the material ‘cherry’ as shown in Figure 1. Both DALL-E 2 and Midjourney v2 depicted the object cherry instead of the cherry wood material. Similar for Ipswich pine. In MERL, it stands for a type of wood, but in Midjourney v2 this text prompt is interpreted, very creatively, as a sphere in front of a pine forest. At the same time, other materials show rather consistent appearances among the three datasets, for instance, black soft plastic and orange paint. Generative models are also known to be more strongly triggered by specific words that were frequently represented in the training set. As a result, visual differences in the images created by these models may simply be due to a higher familiarity with certain words, which can vary across MERL keywords. Note that we deliberately choose not to change the text descriptions from MERL as the prompts, because we do not want to bring in one more uncontrolled variable, which is the deviation or modification of the material descriptions. We are more interested in observing the outputs.

Another interesting observation that can be made from Figure 3 is overall differences in training and cross-validation accuracies, indicating how coherent triplet judgements contribute to the embedding (Künstle et al., 2022). The Midjourney v2 embedding yielded a substantially lower training and cross-validation accuracy than the other two embeddings, indicating a higher noise level. In the context of the current study, noise suggests lapses, imprecision or disagreement between participants. One possible explanation is the Midjourney v2 created unique materials that are different from existing material datasets. The various unique and interesting patterns within the sphere shape might introduce ambiguity, which leads to a higher noise level. As shown in Figure 1, compared with MERL and DALL-E 2, Midjourney v2 has more textures or patterns within the material spheres, as well as more diverse backgrounds. The overall visual style can be described as fantasy like. Note that DALL-E 2, the other generative AI, produced less ambiguity than Midjourney v2. One possible reason is the visual style of DALL-E 2 is truer to life. This diversity between Midjourney v2 and DALL-E 2 has also been observed by Göring, Rao, Merten, and Raake (2023) who concluded that the former has a more artistic style and the latter a more realistic one. (See also the discussion of Experiment 2 with the interpretation of Figure 10.)

**Figure 5.**
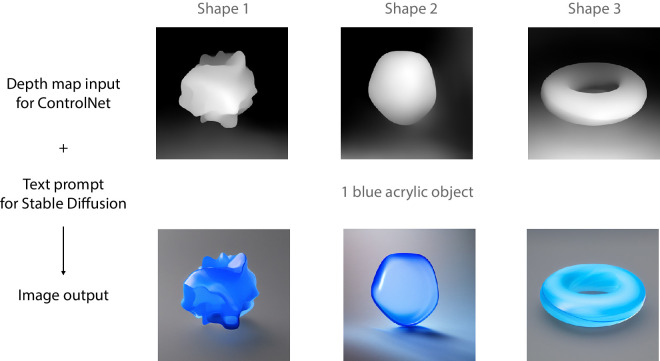
Image generation for Experiment 2, using ControlNet with depth maps and Stable Diffusion v1.5 with text prompts. The top row presents the depth maps of three different shapes, input for ControlNet. The bottom row includes the final images.

**Figure 6.**
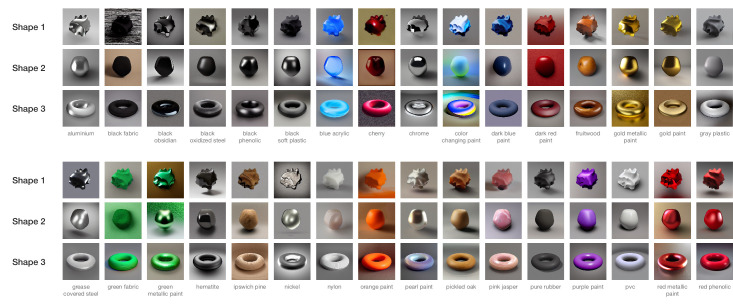
An overview of all 32 x 3 stimuli used in three separate sessions in Experiment 2.

**Figure 7.**
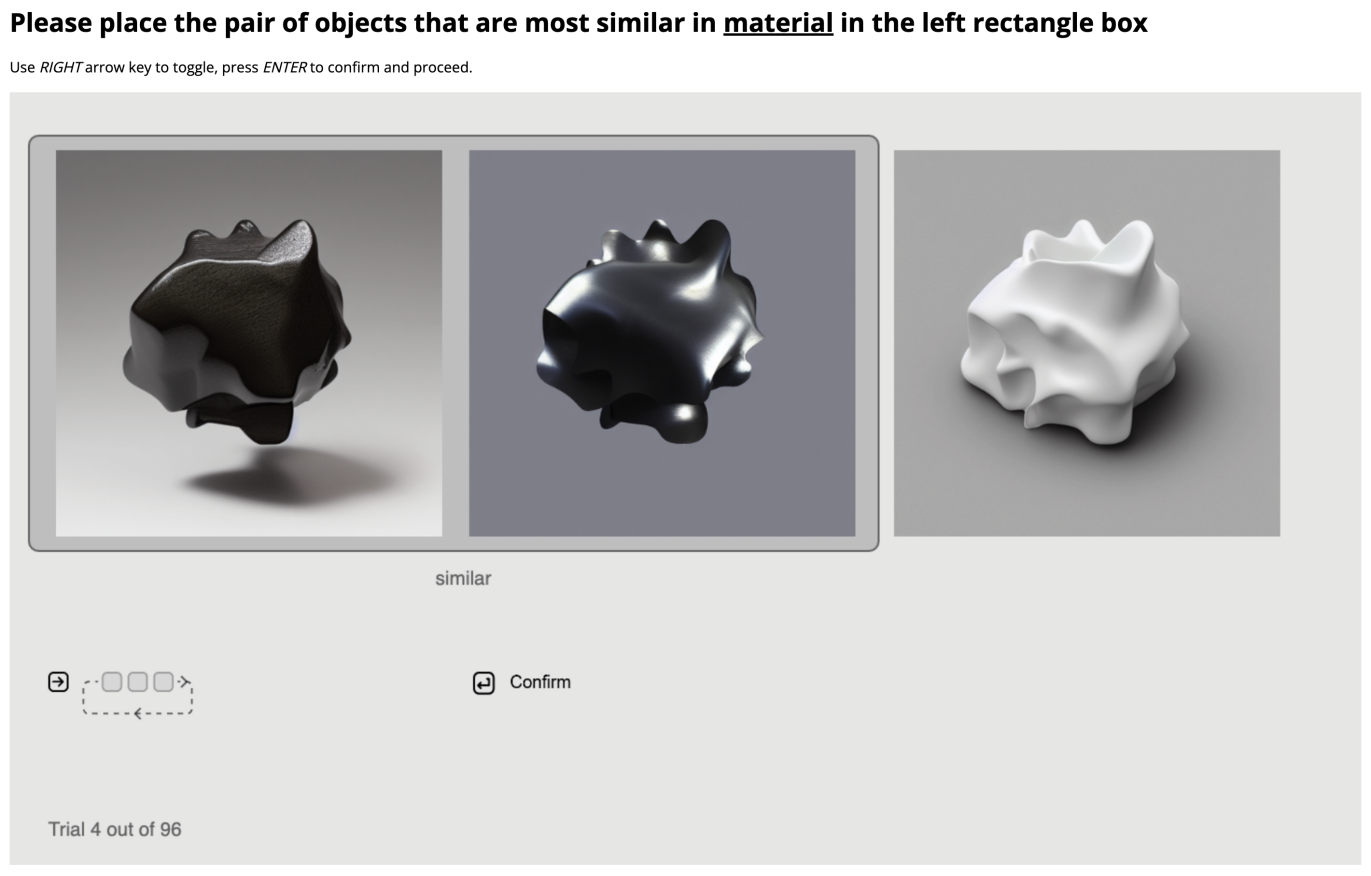
Interface for online Experiment 2. As the icon on the left-hand side indicates, Pressing the RIGHT arrow key would toggle the order of the three images. All three possible pairs can be selected as being similar in terms of material. The participants can press RETURN to both confirm their choice and proceed to the next trial.

**Figure 8.**
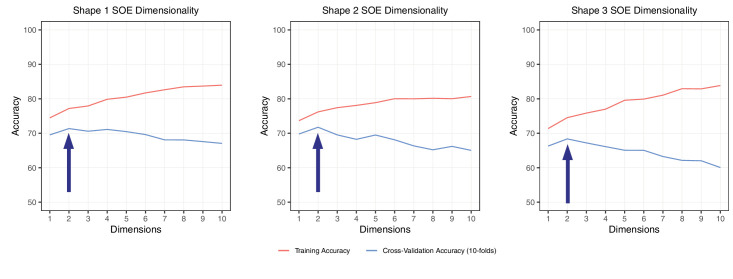
The dimensionality of embeddings of shapes 1 to 3. The peaks of the blue cross-validation curves indicate the optimal dimensionality. All three embeddings yielded a 2D solution.

**Figure 9.**
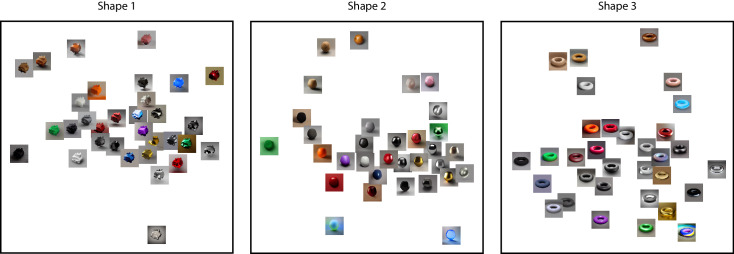
The 2D embeddings of shapes 1 to 3 after Procrustes analysis, the embeddings for shapes 2 and 3 being aligned with that of shape 1.

**Figure 10.**
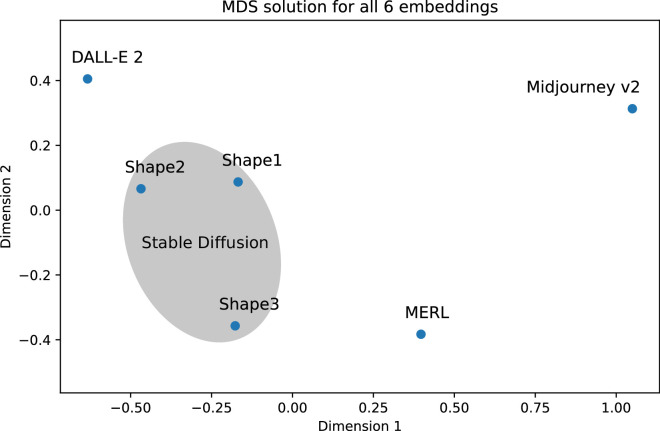
The MDS solution for all six embeddings. The canonical correlation coefficients were used to build the distance matrix.

In summary, in Experiment 1, we used two AI models to generate material images according to text description from a classic BRDF material dataset, MERL. The resulting materials from AI are unique and different, not so comparable with those from MERL. Probably the AI models have quite some freedom in the interpretation and generation. Later in early 2023, we learned a tool that can provide more control over image generation. This allowed us the explore more complex shapes than the spheres from Experiment 1, as we did in Experiment 2.

## Experiment 2: Influence of shape

The generative AI text-to-image model Stable Diffusion (Rombach, Blattmann, Lorenz, Esser, & Ommer, 2022) is regularly being used by artists to generate images. Unfortunately, this model faces the same limitation as DALL-E 2 and Midjourney v2, where the text prompt is the only means of controlling the spatial composition of the output images, which can be insufficient. However, in late February 2023, a new add-on for Stable Diffusion was released: ControlNet (Zhang et al., 2023). This add-on provides various ways of precisely controlling the spatial condition of the output images, such as desired human posture, depth map, and Canny edge.

As stated, before the introduction of ControlNet, one major limitation for AI image generation was that the stimulus shape could be controlled through text prompts only. For example, shapes more complex than a sphere are difficult to describe using only text without introducing ambiguity in either shape or orientation. However, in addition to text, ControlNet can achieve control over the spatial conditions of the output as we show later in Figure 5 and Figure 6. This option afforded us to further explore AI generated materials using more complex shapes than the spheres we used in Experiment 1. Moreover, it provided a means to investigate the potential influence of shape on material perception in the domain of generative AI.

### Methods

#### Stimuli

Compared with Experiment 1, where the image output from AI models is controlled by text prompts only, in Experiment 2, image generation by combining Stable Diffusion v1.5 with ControlNet has one more constraint: the precise control of the shape by using depth maps. Figure 5 presents three examples of different depth maps combined with a single text prompt. ControlNet is a neural network architecture that adds spatial conditioning by locking parameters (layers) within a large text-to-image diffusion model such as Stable Diffusion. The weight of the control can be adjusted to vary the degree in relaxation of locking parameters (Zhang et al., 2023). It should be noted that there is no ground truth in the sense that there are no actual 3D models: ControlNet merely converges to solutions that visually resemble the specific 3D shape used as input. We used the same 32 materials from Experiment 1, with depth maps we generated with the pre-processor of ControlNet to control the shape of the material blobs. The weight for ControlNet 1.0 was set to one (i.e., halfway) for all images. For Experiment 2, we changed the text prompts from ‘sphere’ to ‘object’ (e.g., ‘one gray plastic object’). Figure 6 shows an overview of all 32 × 3 stimuli of Experiment 2. We chose three different shapes, two ‘globular’ shapes of high (shape 1) and low (shape 2) complexity, and a topologically different, more regular shape of a torus (shape 3).

#### Procedure

We used the same approach as in Experiment 1: in 3 sessions, 1 per unique shape, we instructed participants to judge the similarity in materials where each session consisted of 96 trials. The only difference is that, instead of having one fixed target image, participants were now free to choose any pair from the triplet. See Figure 7 for the new interface. We changed the task to reduce noise in the data. We noticed from Experiment 1 that a fixed target sometimes makes the choice more difficult when the fixed target is the odd one. Without a target, participants could freely choose from all three possible pairs. In all other respects, the data analysis was the same as in Experiment 1.

#### Participants

Sixty unique participants were recruited for Experiment 2, 20 participants for each session. We recruited all participants from Prolific with the same prescreen criteria as in Experiment 1. The number of recruited participants was less than in Experiment 1 because we anticipated that the new task would produce slightly less noise. The same server issues caused some data loss. Eventually, we recorded 18, 18, and 13 participants for shapes 1 to 3. Yet, the number of triplets we got was well beyond the minimal requirement.

### Results

Figure 8 shows the dimensionality plots for Experiment 2. All three embeddings suggest a 2D solution. Note that the peaks for cross-validation accuracy were all around 70% and more pronounced than in Experiment 1, indicating a reduction in data noise (Künstle et al., 2022).

Figure 9 shows the three 2D embeddings after Procrustes analysis, the embeddings for shapes 2 and 3 being aligned with that of shape 1. Similarities can already be noticed by observation. To strengthen this observed similarity, we classified the 32 text descriptions from the MERL dataset into the material categories such as wood and metal, following the classification by Fleming et al. (2013). In doing so, about the same clustering can be seen in the three embeddings: three wood materials are positioned in the top left corner and glossy metallic materials in the bottom right side, while matte fabric-like materials show up mainly on the left side.

We also found statistical support for the observed similarity from canonical correlation analysis results, as denoted by Table 2. Each pair of the three embeddings shows high correlations (*r* = 0.815, 0.836, and 0.764) which all are significant (*p* < 0.001).

**Table 2.** Canonical correlation results for Experiment 2.

			Canonical weights			
Space1-space2	Correlation coefficient	*p* value	Space1-X	Space1-Y	Space2-X	Space2-Y
Shape1-Shape2	0.815	0.000	−0.411	0.815	−0.482	0.793
Shape1-Shape3	0.836	0.000	−0.547	0.712	−0.621	0.750
Shape2-Shape3	0.764	0.000	0.572	−0.722	0.548	−0.807

A *p* value of 0.000 means *p* < 0.001. The canonical weights indicate how much each dimension contributes to the overall correlation.

To compare all perceptual embeddings representing the various generative models and the BRDF stimulus set, we combined the results from our two experiments as follows. First, we correlated the 2D embeddings of MERL, DALL-E 2, and Midjourney v2 from Experiment 1 with the three embeddings from Experiment 2. This led to nine extra correlations, where correlations between MERL and the three shape embeddings were all significant (shape 1: *r* = 0.567, *p* = 0.019; shape 2: *r* = 0.619, *p* = 0.004; shape 3: *r* = 0.727, *p* < 0.001). Similarly, all correlations for DALL-E 2 were significant (shape 1: *r* = 0.701, *p* < 0.001; shape 2: *r* = 0.781, *p* < 0.001; shape 3: *r* = 0.567, *p* = 0.024). In contradiction, all correlations for Midjourney v2 were not significant (shape 1: *r* = 0.311, *p* = 0.245; shape 2: *r* = 0.136, *p* = 0.969; shape 3: *r* = 0.204, *p* = 0.807). Second, we combined these nine correlations with the values from Tables 1 and 2 into one correlation matrix on which we performed an MDS analysis using the correlations as similarity measures. The outcome of the MDS analysis can be found in Figure 10 with the smallest/largest distance between two embeddings representing highest/lowest correlation.

### Discussion

All three embeddings from Experiment 2 yielded a clear 2D solution with relatively high correlations with each other. The canonical weights as shown in Table 2 imply that both dimensions contribute about equally to the correlations.

Both consistent dimensionality and high similarity among the three embeddings suggest that the results are non-random and reasonably robust. This also suggests that semi-systematic variations in the combination of predefined geometry with uncontrolled illumination have only minor influence on material appearance and perception. Olkkonen and Brainard (2011) investigated the joint effects of illumination and object geometry on material perception and found strong interactions between them. Because the illumination in the current study was not controlled owing to the nature of generative AI models, the variation in illumination might have interfered with the influence of object geometry. In addition, Vangorp et al. (2007) considered the blob shape (with a gently changing smooth surface) to be one of the best choices for material discrimination. All three shapes used in the current study have relatively smooth surfaces, but none of them has a flat surface, which could also explain the limited influence of object geometry.

Recently, Göring et al. (2023) evaluated the perceived realism and image appeal of 135 AI-generated images created by several text-to-image models, including DALL-E 2, Midjourney, and Stable Diffusion. They found that DALL-E 2 and Midjourney were assessed to generate the most and least realistic images, respectively, with Stable Diffusion in between. The Midjourney images were described as “more artistic similar to a painting.” With respect to image appeal, the images from both DALL-E 2 and Midjourney were judged to be about the same but more appealing than the ones created by Stable Diffusion. The MDS solution as shown in Figure 10 suggests that MidJourney v2 cannot be associated with any of the other embeddings, that MERL and DALL-E 2 come up with different solutions, and that shape 3 seems closest to MERL and shapes 1 and 2 to DALL-E 2. Interestingly, the order of the three AI models on the first dimension appears to be compatible with the realism evaluation from Göring et al. (2023) study, with DALL-E 2 and Midjourney being the most and least realistic and both Stable Diffusion and MERL in between. Looking at the second dimension, the order seems consistent with the appeal judgements from the Göring et al. (2023) study in that both DALL-E 2 and Midjourney v2 have the highest values and Stable Diffusion v1.5 (shape 3) the lowest, together with MERL.

## General discussion

In two experiments, we explored human material perception using generative AI stimuli and compared the perceptual embeddings between three different generative AI models (DALL-E 2, Midjourney v2, and Stable Diffusion v1.5) and one computer rendered BRDF stimulus set (MERL). Unlike the computer rendered material stimuli, the generative AI stimuli are not accompanied by their distal characterization (i.e. reflectance parameters and illumination information) and solely rely on the so-called prompt. This limits the range of psychophysical paradigms available to quantify the perceptual appearances of these artificial objects. We chose to explore perceptual embeddings using SOE (Künstle et al., 2022) and found that in all generative AI experiments, the embedding turned out to be two-dimensional. In contrast, the embedding of the BRDF dataset from which the prompts for the AI models were taken, was one-dimensional.

**Figure 11.**
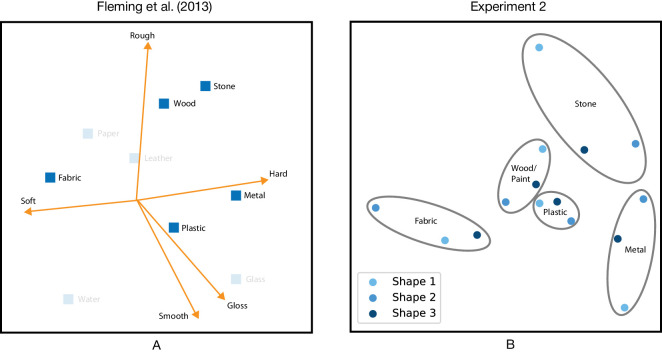
(A) Perceptual material space adapted from Fleming et al. (2013) fitted with attributes vectors. (B) Perceptual space from Experiment 2 with the centroids of five material categories for three shapes.

The difference in dimensions may be attributed to varying levels of stimulus complexity. Although no distal parameters were involved, the generative AI images appeared to contain rich texture information. Some even displayed translucency, a feature absent in the BRDF rendered stimuli owing to their exclusion of subsurface scattering. Interestingly, the *y* axis of the 2D MERL space, as shown in Figure 4 (and its 1D solution), can be loosely associated with one of the main dimensions in the medium-independent material perceptual space (Fleming et al., 2013; Zhang et al., 2019): the opposing hard–soft attributes. Moreover, in Experiment 2 the more complex shapes, in particular shape 1, immediately adds the second orthogonal dimension associated with smooth–rough attributes (Fleming et al., 2013; Zhang et al., 2019), recovering the 2D space of the medium-independent material perception space, as shown in Figure 11.

One difference between Experiments 1 and 2 is the numbers of constraints for generative AI models. In Experiment 1, text description from MERL dataset was the only constraint for the generative AI models, while in Experiment 2, we also introduced a depth map as the second visual constraint in addition to the semantic one. The text descriptions for materials could cause semantic ambiguity for AI models. As we mentioned in the discussion of Experiment 1, both DALL-E 2 and Midjourney v2 could have their own interpretations of the descriptions. Some interpretations were literal, not necessarily correct or wrong. For example, cherry can be both interpreted as wood or fruit. In addition, AI can generate images beyond reality, for example, a single cherry with stem from DALL-E 2 and a bunch of cherries pressed within one sphere from Midjourney v2.

From another angle, the results might reflect the inequality between the two modalities of language and vision. Language has certain limitations when describing some image features, such as complex geometry or spatial information. For instance, the first shape used in Experiment 2 can be challenging to describe with only text, without leaving room for different interpretations and imaginations. Another example is when announcing the exact location or entrance of a conference, text description is often paired with a map or photograph to eliminate ambiguity. Liao, Sawayama, and Xiao (2024) have also reported that the verbal descriptions are unable to convey the visual nuances of material appearances, although they could capture material qualities on the coarse level. This finding also points to the limitation of verbal description.

Although the 2D optimum occurred robustly for every generative AI stimulus set (except the possible six-dimensional solution for DALL-E 2), independent of shape (Experiment 2) or generative model (Experiments 1 and 2), the dimensionality is relatively low in comparison with other material perception studies based on triplet data. For example, Filip, Lukavskỳ, Děchtěrenko, Schmidt, and Fleming (2024) studied the perceptual dimensions of wood, which is only one category in our experiment, and found the optimal number of dimensions to be between five and nine. Next to using a different algorithm, that is, the VICE model by Muttenthaler et al. (2022), they also computed the optimal embedding dimensionality by means of Künstle et al., (2022)’s SOE method and found an optimum at six dimensions. A related study that used a wide variety of photos from the STUFF dataset (Schmidt et al., 2022) revealed that 36 dimensions were needed to describe similarities between material photos.

Hebart et al. (2020) using the ‘THINGS’ dataset instead of the ‘STUFF’ dataset, came up with 49 dimensions. Different dimensionalities may arise from focusing on either a single material category or a diverse range of materials. Each approach can lead to distinct criteria, resulting in unique perceptual spaces with varying dimensions.

Although the dimensionalities vary widely between these studies and ours, it is interesting to see that the accuracies are rather similar. Accuracy means the percentage of raw triplet data being the same to the triplet data predictions that arise from a model or directly from the embedding itself. The lowest accuracy was found in a study with the largest diversity in pictures, that is, the THINGS database, with Hebart et al. (2020) reporting approximately 65% accuracy. Although this appears low, it is high when compared with their upper limit of approximately 67%, which was computed on the bases of repeated trials by different observers. The STUFF database (Schmidt et al., 2022) yielded an accuracy of 71.86% with an upper limit of 73.84%, and the study on wood (Filip et al., 2024) resulted in an accuracy of approximately 76% with an upper limit of 82%. This accuracy is comparable to the 68% to 72% cross-validation accuracy we found in Experiment 2. Note that we did not measure repeated trials and can therefore not compute an upper limit. Experiment 1 yielded a somewhat different picture with 71% cross-validation accuracy for MERL, 70% for DALL-E 2, and 61% for Midjourney v2. Mind that all these accuracies were calculated at the optimal number of dimensions (i.e., two in all our cases except MERL).

In summary, although it is challenging to make direct comparisons between studies owing to differences in dimensionality and methodology, the perceptual embeddings from our synthetic generative AI stimuli have accuracy levels comparable with those of previous studies using photos. Last, it should be noted that Lagunas et al. (2019) also applied a triplet similarity task to their stimulus set. Yet they did not explore the perceptual embedding as a (potentially) interpretable global space.

We are not the first using AI-generated stimuli for research into material perception, being aware of studies using prompt-based diffusion models. These studies used a variational auto-encoder on the perception of glossiness (Storrs, Anderson, & Fleming, 2021) and a generative adversarial network on the perception of translucency (Liao et al., 2023). In both studies, the architectures were specifically trained on predefined image datasets (albeit unsupervised) and with different research scopes from ours. Storrs, Anderson, and Fleming (2021) found that variational auto-encoders clustered glossy and matte objects in a manner similar to humans and proposed that the unsupervised model (as opposed to a supervised model) could well predict human gloss perception. Liao et al. (2023) found that distinct layers in their generative model corresponded with different perceptual attributes, where the middle layers corresponded with translucency and higher layers corresponded with body color. Finding paths in latent space that correspond with the intensity of material attributes brings generative AI images closer to traditional CGI in which, for example, reflectance parameters can be manipulated. Altering material appearance via latent space was also explored by Delanoy, Lagunas, Condor, Gutierrez, and Masia (2022) using generative adverserial networks and by Sharma et al., (2024) using a diffusion model (Stable Diffusion v1.5). Although Delanoy et al. (2022) used the same MERL dataset (Lagunas et al., 2019) as we used, in their study the images were the starting point to generate novel stimuli, whereas in our study BRDF labels were the starting point. Hence, our study complements other studies using generative AI for material perception as we used text-based images and explored their perceptual embeddings.

AI-generated imagery forms a new medium to explore material perception. Using this new medium, we find that our results correspond with studies using real-world photos (Fleming et al., 2013), CGI renderings (Zhang et al., 2019) and paintings (van Zuijlen et al., 2020). This is illustrated in Figure 11 where Figure 11A presents the perceptual material space as found by Fleming et al. (2013), where the first two dimensions may be associated with soft–hard and smooth–rough, and Figure 11B summarizes the three embeddings from Experiment 2 in the form of the five centroids of five material categories. The perceptual space on the left has wood and stone at the top, and from left to right, fabric, plastic and metal in the middle. The space from Experiment 2 on the right side has a similar structure, only the position of plastic seems to be shifted upward. Both Zhang et al. (2019) and van Zuijlen et al. (2020) found similarly structured perceptual spaces as Fleming et al. (2013). Yet it should be noted that we did not have a complete and evenly distributed material category set, because our stimuli were confined to the names of the MERL dataset.

Finally, the word medium can be used to describe not only what the image is made of (e.g., oil paint on canvas, pixels on a screen, or drawing on paper), but also the technique with which the image has been made (e.g., painting, animating, sketching, or photographing). The few studies that directly compared different media yield a mixed picture. Delanoy, Serrano, Masia, and Gutierrez (2021) compared paintings and renderings and found comparable material perceptions across these two media, whereas Zhao, Stumpel, de Ridder, and Wijntjes (2024) compared paintings with engravings and did find differences that could be attributed mainly to contrast. In the latter study, the lack of color seemed to have been (over)compensated through additional local contrast applied by engravers. Generative AI is clearly not a traditional medium and does not depend as much on the interaction between artist and material in the same way as rendering, painting, and engraving. Although it appears a fundamentally distinct medium, it does have similarities. The role of the artist or creator is conceptually similar, but the practice of handling the brush is transitioned into handling the prompt. Another similarity with other media is that it has limitations, and these limitations may lead to specific creativity. One of the limitations of generative AI is its stochastic nature: the relation between prompt and image is rather undeterministic and it is impossible to control every pixel of the image. So the unexpected findings might invite serendipity, probably more so than the traditional act of image making.

As for future steps, we acknowledge that Experiment 2 introduced two changes compared with Experiment 1: a different generative model and shape control via ControlNet. As a result, it is difficult to conclude whether the improved consistency and robustness of the resulting perceptual spaces come from which factor, or a combination of both. Hence for future studies, we suggest to repeat our Experiment 1 using Stable Diffusion v1.5, so that it is more comparable with Experiment 2. Another benefit of using Stable Diffusion, an open-source model, is reproducibility. DALL-E and Midjourney, on the other hand, can be taken offline or updated to reduce reproducibility. In addition, another direction for future research is to collect human judgements on perceived material category, physical accuracy, or other material properties such as glossiness. The judgment data can then be used to further explore the perceptual spaces of generative AI images.

Discussing future work is challenging given the disparity between the pace of vision science publication and the rapid development of generative AI models. In our study, we used algorithms that are (partially) superseded by new ones. Beside the clearly negative aspects of coping with a moving target, a positive aspect may be that our study already has historic relevance, which is why we reported the dates on which the stimuli were generated. Given the uncertain value of future speculation, we do want to highlight one direction that is likely to remain relevant: prompt engineering. In our study, we took a somewhat extreme position by minimising the role of prompting by literally using the MERL labels. This resulted in some atypical stimuli like the cherry ball, where the original label referred to cherry wood. We did that on purpose because choosing otherwise would inevitably lead to subjective choices about how materials should be described, which seems to merit its own study. The choices underlying prompts used to generate images, prompt engineering, will definitely be an interesting playing ground for vision scientists in the coming years.

To summarize, we evaluated the visual output of AI generative models using identical material prompts taken from MERL, a BRDF dataset. To this end, we compared their perceptual spaces derived from triplet similarity judgments. In the first experiment, the perceptual spaces of DALL-E 2 and Midjourney v2 turned out to be unrelated, suggesting that these models have different styles in realistically visualizing materials, in line with earlier observations on perceived realism and appeal of AI generative models (Göring et al., 2023). So, like painters choosing the medium (oil paint, pencil, charcoal, etc.) to visualize materials, it seems wise to do the same when selecting the most appropriate AI generative model. The results of the second experiment indicating minor influence of shape on material representation suggest that this choice does not depend critically on the object’s shape. In this experiment, the shape was controlled by combining the open-source text-to-image AI model Stable Diffusion v1.5 with ControlNet allowing the additional constraints of depth maps. The resulting perceptual space showed not only a similar structure as the MERL embedding, but was also like perceptual spaces from other material studies using real-world photos, computer renderings and depictions. So, generative AI models have unlocked new methods to generate images. Our comparative study has made clear that they may indeed provide a rich and valuable source for the production of visual stimuli to study material perception.

## Footnotes

1 We initially were under the impression that the embedding algorithm would only be able to process comparisons with the middle target, instead of choosing the more intuitive ’odd one out.’ Later we learned that SOE *was* able to process this type of data, which we then used in Experiment 2.
